# Modified SVPWM technique for CMV reduction in asymmetrical dual three phase induction machine drive

**DOI:** 10.1038/s41598-023-48339-3

**Published:** 2023-12-13

**Authors:** Manoj K. Chaudhury, Mukesh K. Pathak, Girish K. Singh

**Affiliations:** Department of Electrical Engineering, IIT Roorkee, Roorkee, India

**Keywords:** Electrical and electronic engineering, Energy infrastructure

## Abstract

Due to its advantages, the asymmetrical dual three-phase induction motor drive is a strong choice in high-power applications. However, the common-mode voltage produced by the voltage source inverters affects the winding insulation and damages the bearings. Common-mode voltage is also responsible for electromagnetic interference and leakage currents. This paper, therefore, analyses the common-mode voltage produced by the inverter supplying a dual three-phase induction motor drive and proposes a novel modified space vector decomposition-based Space Vector Pulse Width Modulation (SVPWM) technique for common mode reduction. The vector space decomposition-based space vector modulation technique offers excellent flexibility as it reduces the common-mode voltage (CMV) by exploiting the additional degree of freedom in a dual three-phase system. The common-mode voltage (CMV) can be reduced to one-sixth of the DC link voltage compared to the highest CMV, i.e. half of the DC-link voltage produced in conventional space vector modulation. The proposed method is also validated experimentally to demonstrate the effectiveness of the proposed scheme in terms of the amplitude of CMV, pulsations, and total harmonic distortion(THD) in current.

## Introduction

Dual three-phase adjustable speed drives are becoming popular in recent times due to various advantages in comparison with conventional three-phase drives^1^. Higher reliability, reduced switch rating, fault tolerance capability^2^, and reduced torque ripple^3^ etc. are some of the advantages offered by the dual three-phase machine. Voltage source inverters are extensively used for variable speed drives involving dual three-phase machines; however, the issues of CMV do exist in multi-phase machines^4,5^ for industrial applications. CMV produces common-mode currents that lead to bearing damage^6,7^, results in electromagnetic interference^8^, and also injures the winding insulation. CMV produces common-mode currents that lead to bearing damage due to high dv/dt at the motor terminals; common-mode currents in such circumstances include electrostatic discharge machining (EDM) currents, circulating currents, and rotor ground currents. The two solutions now available for three-phase systems to solve the above issue are (i) hardware and (ii) software solutions. Hardware solutions include (a) common-mode chokes, (b) passive and active filters, and (c) various inverter topologies. These techniques can partially solve the CMV issue, but they can increase size, weight, expense, and complexity. On the other hand, software solutions that modify switching signals or modulation techniques tend to be more effective. Building software solutions is much simpler since highly effective Digital Signal Processors (DSPs) and Field Programmable Gate Arrays (FPGAs) controllers are readily available..

The concept behind CMV reduction is that active voltage vectors can eliminate zero voltage vectors in a three-phase machine fed by either a two-level or multi-level inverter^9–11^. Common-mode voltage reduction using the active zero state for dual three-phase induction motor drive is mentioned in^12^, where simulation results are presented showing that the common-mode voltage can be reduced by extending the active zero state SVPWM in three-phase inverters to six-phase inverters. In the case of a five-phase induction motor drive, the open-end winding setup for CMV reduction is a straightforward extension of the three-phase idea^13^. In the case of dual three-phase inverters, CMV reduction approaches that are based on carrier-based PWM (CPWM) are utilized in order to lower the CMV; nevertheless, this results in a higher level of current distortion. A generalized strategy for the reduction of CMV is based on reference order and is applicable to an odd number of phases^14,15^. The reduction of CMV for five-phase induction motor drives employing SVPWM in the overmodulation region is mentioned in Refs.^16,17^. Performance of the six-phase induction machine with active voltage vector-based direct torque control to reduce the common-mode voltage is found in Ref.^18^. The vector space decomposition (VSD) method for the dual three-phase machine is introduced in Ref.^19^. This method offers a great deal of simplicity in terms of ease of controlling the machine, reducing the number of sensors used for the closed-loop control. The additional degree of freedom available with the VSD-based space vector modulation is being exploited here to minimize the CMV in the case of a dual three-phase induction machine (DTIM). One of the significant advantages of the VSD method is that the control techniques can be implemented quickly. The six-phase voltages, when transformed to three mutually perpendicular surfaces, the components in the $\alpha -\beta$ plane are responsible for electromagnetic energy conversion. The components on $\mu _1-\mu _2$ subspace contribute to harmonics in the system. The components on $z_1-z_2$ subspace contain zero sequence components. Therefore, the reference vector is synthesized so that the components in $\mu _1-\mu _2$ subspace are kept minimal.

The dual three-phase induction machine has isolated neutrals. So, the components of currents in $z_1-z_2$ subspace remain zero. So it has one set of $\alpha -\beta$ currents like its three-phase counterparts, which is the main advantage of the VSD-based approach. Therefore, an attempt is made in this article to develop a modulation technique for reducing the common-mode voltage using the VSD approach.

The DTIM fed from two three-phase inverters is studied first with conventional SVPWM and implemented using the VSD approach. Then a pair of active voltage vectors are chosen on the $\alpha \beta$-plane of the VSD approach to replace the null vector. A comparison is made between the two approaches to verify the merits of the proposed approach over the conventional method^20–22^. The highest CMV-producing switching states are excluded in the proposed approach. In a two-level three-phase inverter, there are two such states, namely $V_0$ and $V_7$, which have a CMV of $\frac{V_{dc}}{2}$. Similarly, in a six-phase inverter, a pair of null vectors exists, which are replaced by a pair of active vectors depending on the position of the desired space vector on the dq-plane. Following are the contributions of this paper.

- Vector space decomposition theory based SVPWM technique is developed for CMV reduction in inverter-fed dual three-phase induction machine.
- The proposed work addresses the amplitude and pulsation in the common-mode voltage and reduces both.
- The switching states are selected so that it results in minimum current components in the $\mu _1-\mu _2$ plane. Thus the losses are kept to a minimum.

This paper is structured into four sections. Section "Methodology" includes the VSD-based conventional space vector modulation technique and the proposed space vector modulation technique for the dual three-phase machine. Section "Result and discussion" consists of simulation and hardware implementation for the proposed scheme, and Sect. "Conclusion" presents the concluding remark. The modeling of the dual three-phase machine modeling using the vector space decomposition technique and is mentioned in Appendix.

## Methodology

The vector space decomposition method is a powerful tool for the dual three-phase machine. In this approach, the six-dimensional space is converted into three orthogonal subspaces ($\alpha ,\beta$), $(\mu _1,\mu _2)$ and $(z_1,z_2)$^23^. The components on the ($\alpha ,\beta$) plane are corresponding to the electromechanical energy conversion. The other four components are not involved in the energy conversion process. But at the same time, the components on $(\mu _1,\mu _2)$ subspace produce harmonics, and those on $(z_1,z_2)$ subspace produce the zero sequence currents.

**Figure 1:**
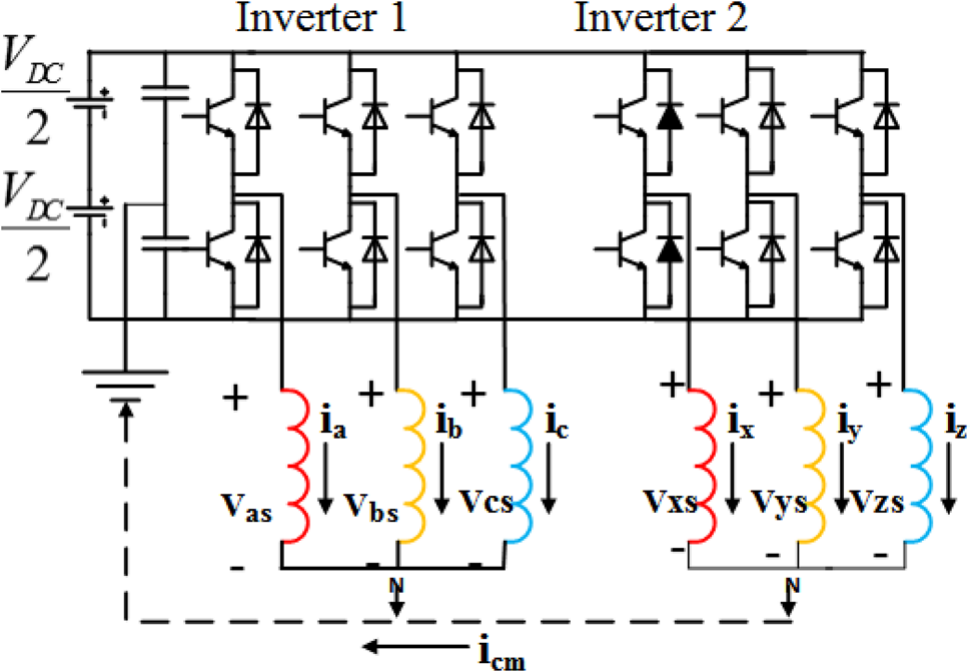
Dual three-phase induction motor drive fed from two-Level inverter with single DC link.

**Figure 2:**
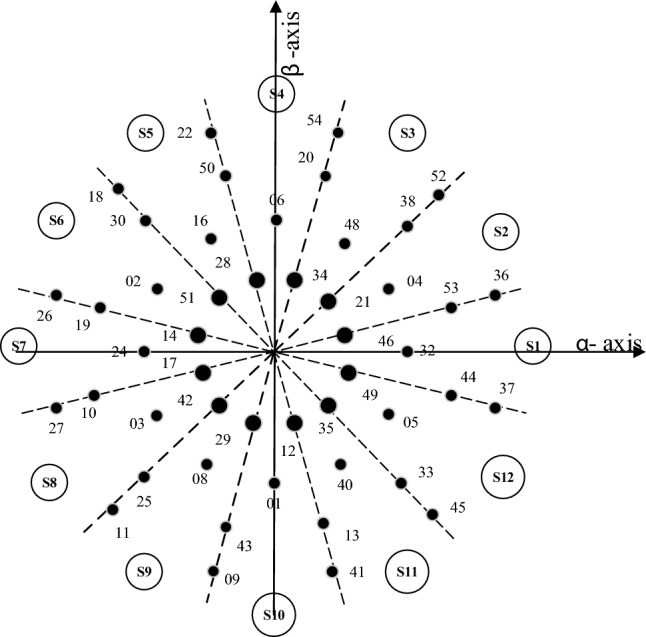
12-sector space vector diagram representing Voltage vectors in $\alpha$ $\beta$ plane.

**Figure 3:**
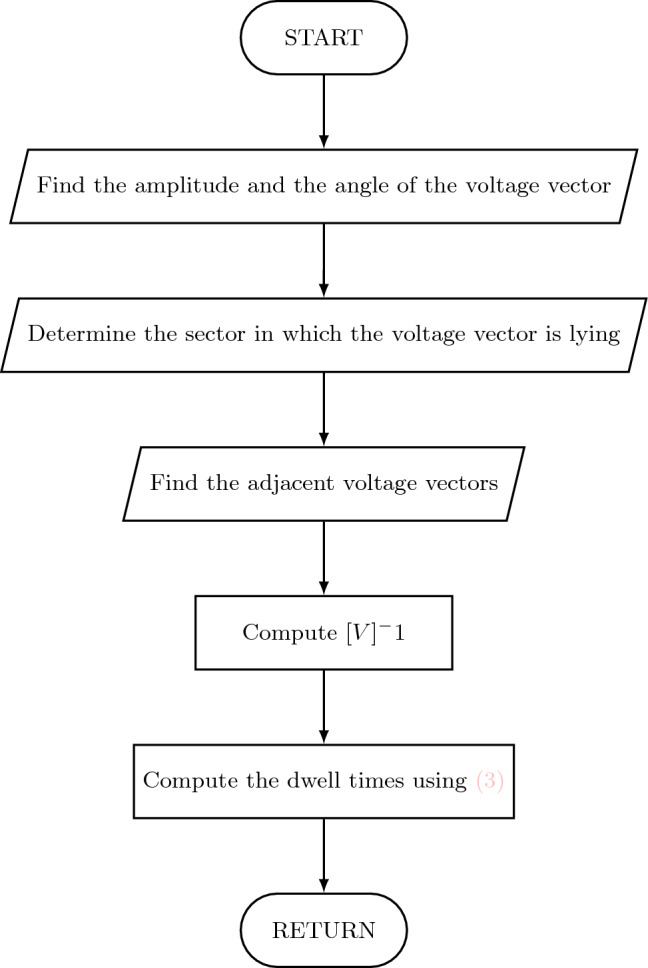
Flow chart for dwell time calculation.

The six-phase inverter (Fig. 1) can be considered as two three-phase inverters sharing a common DC link. Subsequently, the phase voltages can be expressed as:

1 $$\begin{aligned} \begin{bmatrix} V_{as}\\ V_{bs}\\ V_{cs}\\ V_{xs}\\ V_{ys}\\ V_{zs}\\ \end{bmatrix}=\begin{bmatrix} \frac{2}{3} &{} \frac{-1}{3}&{} \frac{-1}{3} &{} 0 &{} 0 &{} 0\\ \frac{-1}{3} &{} \frac{2}{3}&{} \frac{-1}{3} &{} 0 &{} 0 &{} 0\\ \frac{-1}{3} &{} \frac{2}{3}&{} \frac{-1}{3} &{} 0 &{} 0 &{} 0\\ 0 &{} 0 &{} 0 &{} \frac{2}{3} &{} \frac{-1}{3}&{} \frac{-1}{3} \\ 0 &{} 0 &{} 0 &{} \frac{-1}{3} &{} \frac{2}{3}&{} \frac{-1}{3}\\ 0 &{} 0 &{} 0 &{} \frac{-1}{3} &{} \frac{-1}{3}&{} \frac{2}{3}\\ \end{bmatrix}.\begin{bmatrix} S_a\\ S_b\\ S_c\\ S_x\\ S_y\\ S_z \end{bmatrix}.V_{DC} \end{aligned}$$

where s refers to stator and S refers to switching vector of the corresponding inverter leg

By using the vector space decomposition method, the voltage vectors in ($\alpha ,\beta$), $(\mu _1,\mu _2)$, and $(z_1,z_2)$ plane can be obtained easily. The objective of the space vector PWM technique is to apply the $\alpha -\beta$ component of the stator voltage vector duly produced by the control system. The average voltage vectors produced in the other two planes should be kept at a minimum to reduce large harmonic currents.

The voltage vectors in each sector and their timings can be decided by solving a few equations. This involves two equations each for obtaining the voltage vector in $\alpha -\beta$ reference frame (I), zero voltage vector in $\mu _1-\mu _2$ and $z_1-z_2$ plane (II), and one equation for defining the time period (III).

As neutral points of the machine are isolated, there is no current in the $z_1-z_2$ plane. So to implement any SVPWM technique, five equations are to be solved. These equations are:

2 $$\begin{aligned} {\left\{ \begin{array}{ll} \sum \limits _{j=1}^{n} V_k^{\alpha }t_k = V_{s\alpha }^*T_s\\ \sum \limits _{j=1}^{n} V_k^{\beta }t_k = V_{s\beta }^*T_s\\ \sum \limits _{j=1}^{n} V_k^{\mu _1}t_k = 0\\ \sum \limits _{j=1}^{n} V_k^{\mu _2}t_k = 0\\ \sum \limits _{j=1}^{n} t_k =T_s\\ \end{array}\right. } \end{aligned}$$

where *n* denotes the number of voltage vectors used to synthesize the reference in any sector. $V_k^x$ and $t_k$ are the projection of $k^{th}$ voltage vector on the *x*-axis and $T_s$ is the dwell time of a particular voltage vector corresponding to the switching sequence. The reference voltage vector is generated in a 12 sector $\alpha -\beta$ plane (Fig. 2) as discussed below:

The steps for finding the dwell times in each sector involve some calculation as given in a flow chart in Fig. 3. The matrix V comprises of the components of the voltage vectors in $\alpha -\beta$, $\mu _1-\mu _2$ plane along with the dwell times. Equation (2) can written as:

3 $$\begin{aligned} \begin{bmatrix} V_1^{\alpha } &{} V_2^{\alpha }&{} V_3^{\alpha } &{} V_4^{\alpha } &{} V_5^{\alpha }\\ V_1^{\beta } &{} V_2^{\beta } &{} V_3^{\beta } &{} V_4^{\beta } &{} V_5^{\beta }\\ V_1^{\mu 1} &{} V_2^{\mu 1} &{} V_3^{\mu 1} &{} V_4^{\mu 1} &{} V_5^{\mu 1}\\ V_1^{\mu 2} &{} V_2^{\mu 2} &{} V_3^{\mu 2} &{} V_4^{\mu 2} &{} V_5^{\mu 2}\\ 1 &{} 1 &{} 1 &{} 1 &{} 1\\ \end{bmatrix}.\begin{bmatrix} t_1\\ t_2\\ t_3\\ t_4\\ t_5 \end{bmatrix}=[V].\begin{bmatrix} t_1\\ t_2\\ t_3\\ t_4\\ t_5 \end{bmatrix}=\begin{bmatrix} V_{s\alpha }^*\\ V_{s\beta }^*\\ 0\\ 0\\ 1 \end{bmatrix}.T_s \end{aligned}$$

**Figure 4:**
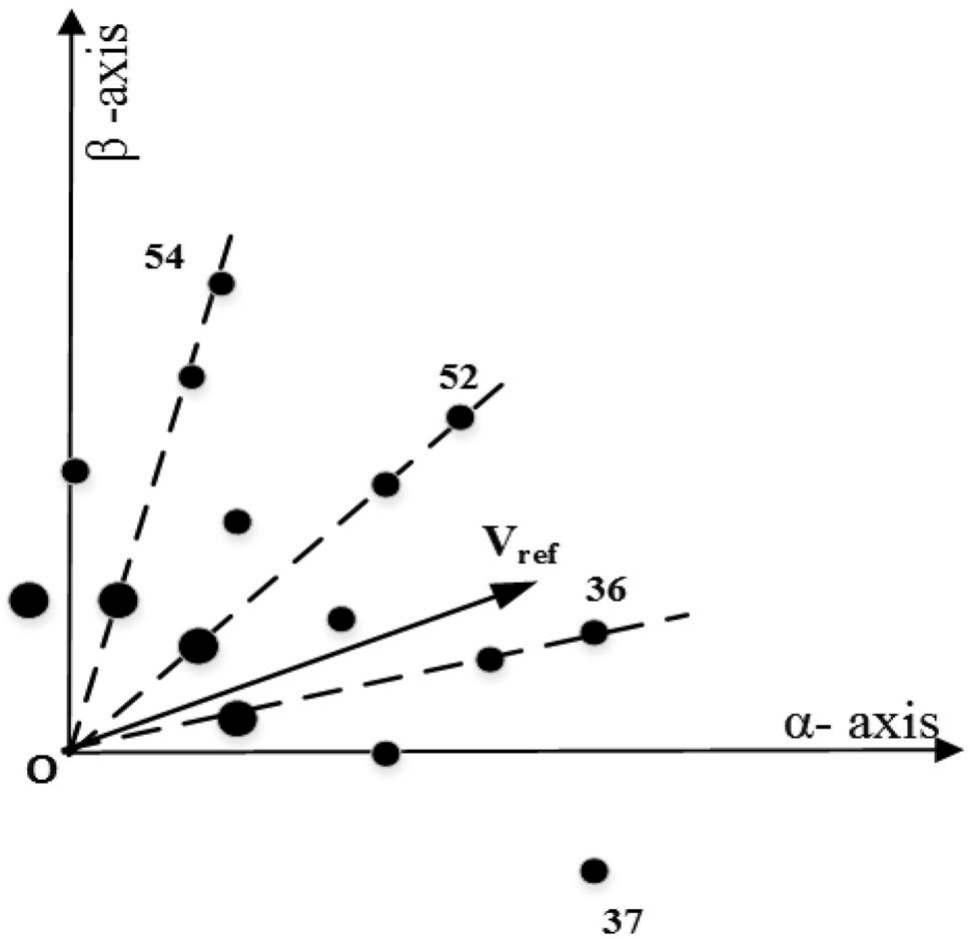
Voltage vectors in sector-I.

**Figure 5:**
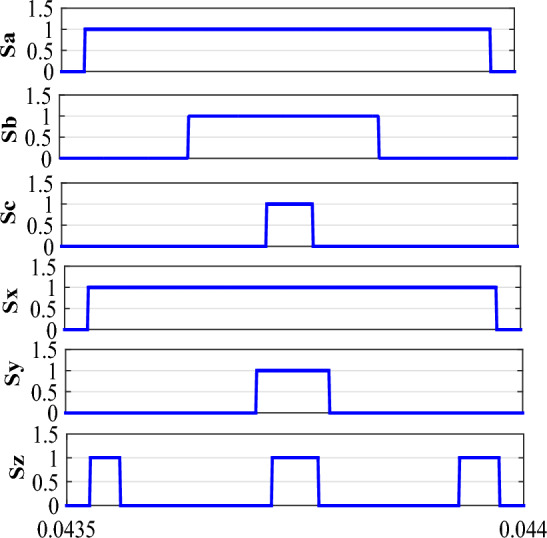
Switching signals in sector-II.

The reference voltage vector is synthesized using the five voltage vectors namely 36, 37, 52, 54, 0 as mentioned below when in sector-I (Fig. 4). Similar to the conventional SVPWM, to have symmetry, the switching sequence is *V*(0), *V*(37), *V*(36), *V*(52), *V*(54), *V*(52), *V*(36), *V*(37), *V*(0). The switching signals are shown in Fig. 5.

### Proposed SVPWM for common-mode voltage reduction

Common mode voltage (CMV) is defined as the potential of the load neutral to earth (in this case, the DC bus midpoint). CMV in the case of an n-phase voltage source inverter, is given by Eq. (4) and the CMV produced by the two three-phase inverter is given by Eqs. (5) and (6).

4 $$\begin{aligned}{} & {} {[}t!] V_{CMV}=\frac{1}{n}\sum \limits _{i=1}^{n} V_{oi} \end{aligned}$$

5 $$\begin{aligned}{} & {} \begin{aligned} V_{CM}\cong V_{no}&=\frac{V_{ao}+V_{bo}+V_{co}}{3}\\&=\frac{V_{dc}}{3}.(S_a+S_b+S_c)-\frac{V_{dc}}{2} \end{aligned} \end{aligned}$$

Similarly, for the second inverter, the CMV produced is;

6 $$\begin{aligned} \begin{aligned} V_{CM}\cong V_{no}&=\frac{V_{xo}+V_{yo}+V_{zo}}{3}\\&=\frac{V_{dc}}{3}.(S_x+S_y+S_z)-\frac{V_{dc}}{2} \end{aligned} \end{aligned}$$

where S refers to the switching vector of the respective invertlegs

**Table 1:** Table for switching vector selection.

Sectors	Voltage vectors used without CMV reduction	Voltage vectors used with CMV reduction
I	V(0),V(45),V(37), V(36),V(52)	V(49),V(45),V(37), V(36),V(52),V(14)
II	V(0),V(37),V(36), V(52),V(54)	V(21),V(37),V(36), V(52),V(54),V(42)
III	V(0),V(36),V(52), V(54),V(22)	V(21),V(36),V(52), V(54),V(22),V(42)
IV	V(0),V(52),V(54), V(22),V(18)	V(28),V(52),V(54), V(22),V(18),V(35)
V	V(0),V(54),V(22), V(18),V(26)	V(28),V(54),V(22), V(18),V(26),V(35)
VI	V(0),V(22),V(18), V(26),V(27)	V(14),V(22),V(18), V(26),V(27),V(49)
VII	V(0),V(18),V(26), V(27),V(11)	V(14),V(18),V(26), V(27),V(11),V(49)
VIII	V(0),V(26),V(27), V(11),V(9)	V(42),V(26),V(27), V(11),V(9),V(21)
IX	V(0),V(27),V(11), V(9),V(41)	V(42),V(27),V(11), V(9),V(41),V(21)
X	V(0),V(11),V(9), V(41),V(45)	V(35),V(11),V(9), V(41),V(45),V(28)
XI	V(0),V(9),V(41), V(45),V(37)	V(35),V(9),V(41), V(45),V(37),V(28)
XII	V(0),V(41),V(45), V(37),V(36)	V(49),V(41),V(45), V(37),V(36),V(14)

**Figure 6:**
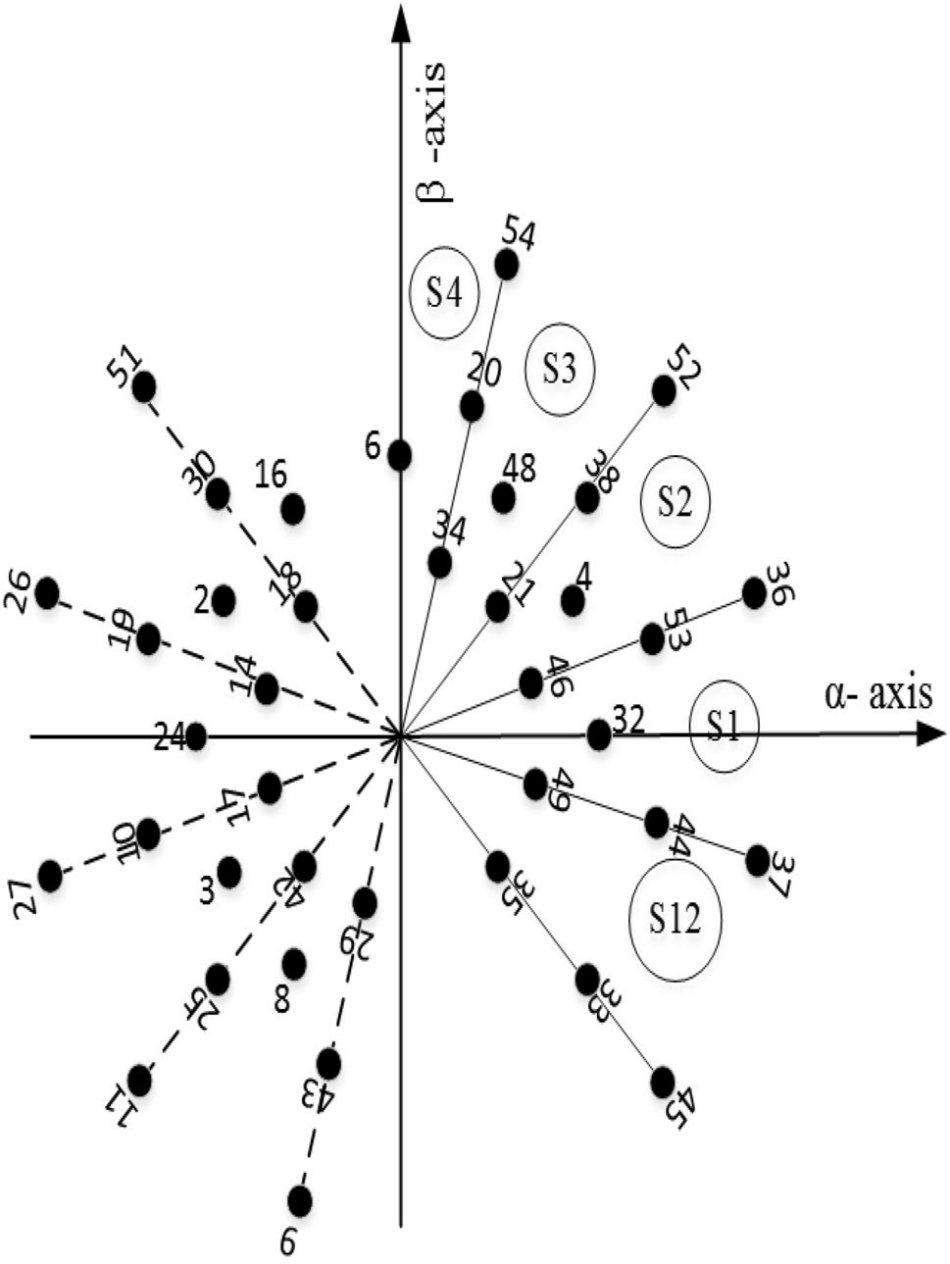
Voltage vectors in sector-I for RCMV.

The vector space decomposition method offers great flexibility in terms of selecting a switching scheme that reduces the CMV. As seen from Fig. 6, in sector-I, the switching sequence with reduced CMV may be selected as *V*(45), *V*(37), *V*(36),*V*(52) and a virtual zero vector comprising of *v*(49), *v*(14). Similarly, in other sectors, the virtual zero voltage vectors used are mentioned below.

The virtual vectors chosen for CMV reduction are not limited to the listed ones only. For example, in sector-I, the virtual zero vector could be any one of the following:

$$V(49)-V(14),V(44)-V(19),V(37)-V(26),V(32)-V(24),$$

$$V(46)-V(17),V(53)-V(10),V(36)-V(27), V(4)-V(3),$$

$$V(21)-V(42), V(38)-V(25),V(52)-V(11),V(48)-V(8), $$

$$V(34)-V(29),V(20)-V(43), V(54)-V(9)$$.

**Figure 7:**
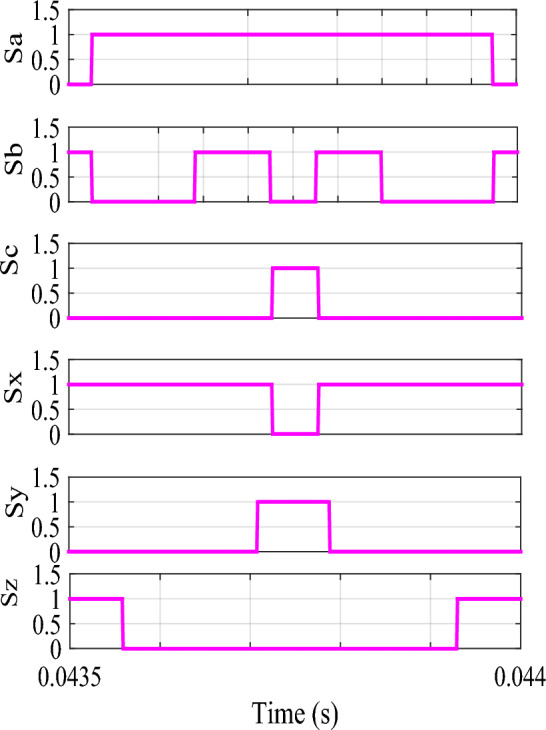
Switching signals in sector-II for RCMV.

The voltage ripple will be minimal if the voltage vectors selected for producing the desired fundamental voltage are close to the reference voltage. The option to choose the virtual voltage vectors in sector-I is shown in Fig. 6. Of course, an optimal switching pattern can be obtained in terms of minimum THD and Switching losses. Based on the above fact, $V(49)-V(14)$ in sector-I is used here for this purpose. The switching signals for the proposed method are shown in Sector-II (Fig. 7). Vectors used in each sector for reduced CMV are mentioned in Table 1.

It is a well-known fact that, in the case of two-level three-phase VSI, CMV produced is $\frac{V_{DC}}{2}$^24^. The dual three-phase induction machine though a six-phase machine, has two isolated neutral. The advantage of such a configuration is well documented. So CMV produced, in this case, is also $\frac{V_{DC}}{2}$. By using the above-proposed algorithm, CMV is limited to $\frac{V_{DC}}{6}$ as the machine has two isolated neutrals. CMV produced by the two-level voltage source inverter is given in Table 2.

**Table 2:** CMV and phase voltage in different states.

State	$V_{ao}$	$V_{bo}$	$V_{co}$	$V_{CM}$
$V_0(000)$	$\frac{-V_{dc}}{2}$	$\frac{-V_{dc}}{2}$	$\frac{-V_{dc}}{2}$	$\frac{-V_{dc}}{2}$
$V_1(100)$	$\frac{V_{dc}}{2}$	$\frac{-V_{dc}}{2}$	$\frac{-V_{dc}}{2}$	$\frac{-V_{dc}}{6}$
$V_2(110)$	$\frac{V_{dc}}{2}$	$\frac{V_{dc}}{2}$	$\frac{-V_{dc}}{2}$	$\frac{V_{dc}}{6}$
$V_3(010)$	$\frac{-V_{dc}}{2}$	$\frac{V_{dc}}{2}$	$\frac{-V_{dc}}{2}$	$\frac{-V_{dc}}{6}$
$V_4(011)$	$\frac{-V_{dc}}{2}$	$\frac{V_{dc}}{2}$	$\frac{V_{dc}}{2}$	$\frac{V_{dc}}{6}$
$V_5(001)$	$\frac{-V_{dc}}{2}$	$\frac{-V_{dc}}{2}$	$\frac{V_{dc}}{2}$	$\frac{-V_{dc}}{6}$
$V_6(101)$	$\frac{V_{dc}}{2}$	$\frac{-V_{dc}}{2}$	$\frac{V_{dc}}{2}$	$\frac{V_{dc}}{6}$
$V_7(111)$	$\frac{V_{dc}}{2}$	$\frac{V_{dc}}{2}$	$\frac{V_{dc}}{2}$	$\frac{-V_{dc}}{2}$

### Performance of the proposed SVPWM technique

#### Number of switching and vector sequence

The proposed reduced common-mode voltage space vector modulation technique (RCMV-SVPWM) is based on the vector space decomposition method. The proposed method is also valid for any number of phases; and, therefore, helps in reducing the common-mode voltage in VSI-fed dual three-phase drives. The switching frequency in the case of RCMV-SVPWM does not remain the same as in the case of conventional SVPWM. But as the switching frequency used is low, particularly for the dual three-phase drives, the increase in switching frequency will not affect much in terms of the switching loss.

#### CMV elimination

The new modulation technique reduces the CMV to one-sixth of the DC link voltage from half of the DC link voltage as found in the case of conventional SVPWM. The switching vectors chosen by the new method result in a lower CMV; nevertheless, when dead times are considered, pulses in the neutral voltage with an amplitude of $\frac{V_{dc}}{2}$ are produced during some of the vector transitions. This undesirable effect has been reported in Refs.^25^ and^26^. Even then, the CMV produced by the RCMV-SVPWM is much less than that produced by the conventional SVPWM.

## Result and discussion

The performance of the proposed RCMV-SVPWM scheme is presented for a six-phase asymmetrical induction motor as load fed from a two-level voltage source inverter. The machine parameters are given in Table 3.

**Table 3:** Machine parameter.

Stator resistance ($$R_{s1}, R_{s2} $$)	$$2.19\Omega $$
Rotor resistance ($R_{r}$)	$4.1 \Omega$
Stator leakage inductance ($L_{ls}$)	0.015*H*
Rotor leakage inductance ($L_{lr}$)	0.015*H*
Magnetizing inductance ($L_m$)	0.119*H*
No. of pole pairs (*p*)	3
Moment of inertia(*J*)	$0.001 kgm^2$

### Simulation result discussion

**Figure 8:**
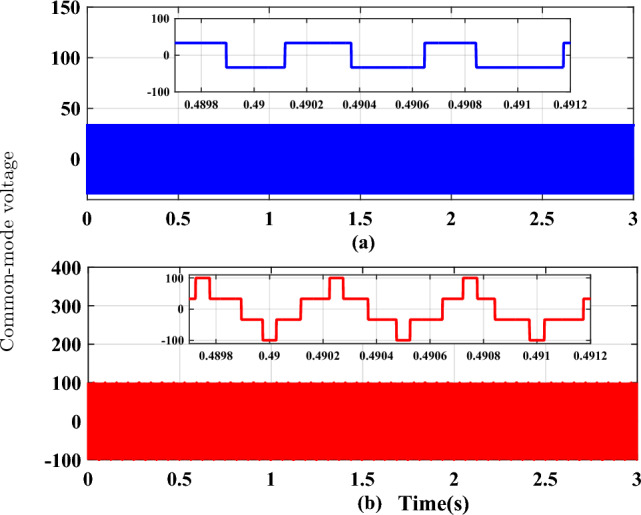
Common mode voltage waveforms (**a**) RCMV-SVPWM, (**b**) SVPWM.

**Figure 9:**
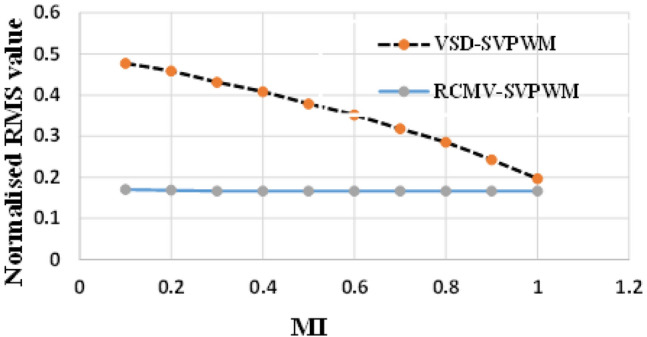
rms common-mode voltage.

**Figure 10:**
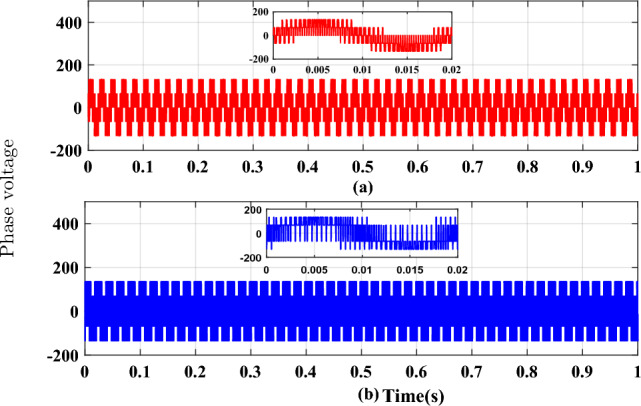
Phase voltage waveforms (**a**) SVPWM, (**b**) RCMV-SVPWM.

**Figure 11:**
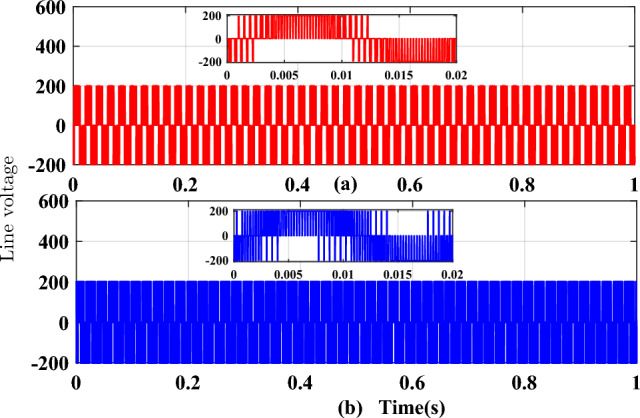
Line voltage waveforms (**a**) SVPWM, (**b**) RCMV-SVPWM.

**Figure 12:**
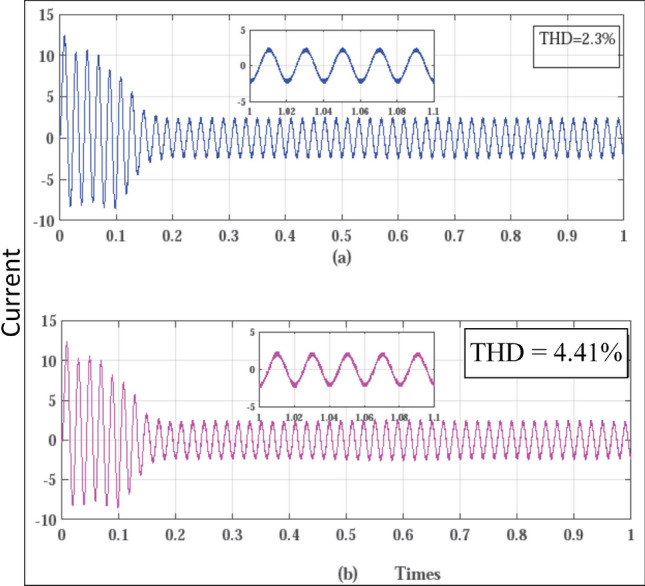
Line current waveforms (**a**) SVPWM, (**b**) RCMV-SVPWM.

**Figure 13:**
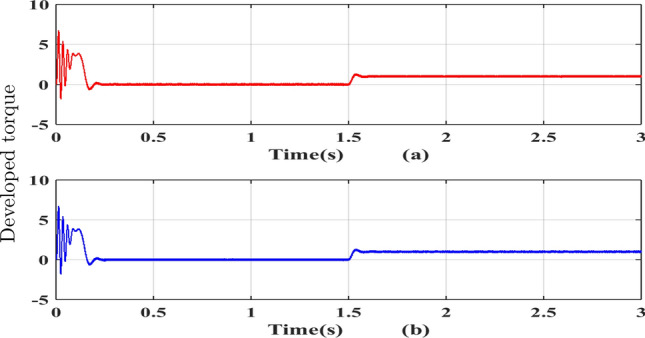
Developed torque (**a**) SVPWM, (**b**) RCMV-SVPWM.

The proposed methodology is simulated on the Matlab platform. A DC link voltage of 200V and a switching frequency of 2kHz is used for the simulation purpose. From the simulation result, it can be observed that the peak amplitude of common-mode voltage generated with the proposed modulation scheme is 33.33 V (Fig. 8a), where the common-mode voltage produced in the conventional SVPWM scheme is 100V (Fig. 8b). The number of pulsations in the common-mode voltage is 6 in the proposed method. The pulsation in the common-mode voltage is 18 in the case of conventional SVPWM, as shown in the zoomed window (8) for each case. Additionally, the RMS value of the common-mode voltage is also reduced by a factor of 1/3. The combined effect results in a decrease of the current due to common-mode voltages by many folds, and hence improve the life of machine insulation and bearing, etc. Figure 9 shows the CMV (RMS value) variation to modulation index for the two cases. It is observed that there is no significant change in the RMS value of common-mode voltage in the proposed scheme, whereas, in a conventional modulation scheme, the variation is from 0.5 pu to 0.2 pu. The output phase voltage waveforms for the conventional SVPWM and the proposed RCMV-SVPWM are shown in Fig. 10. The corresponding line voltage waveforms are shown in Fig. 11. As shown in the zoomed window of one cycle of line voltage, the voltages are primarily similar except for the appearance of very few pulses of opposing polarity.

**Figure 14:**
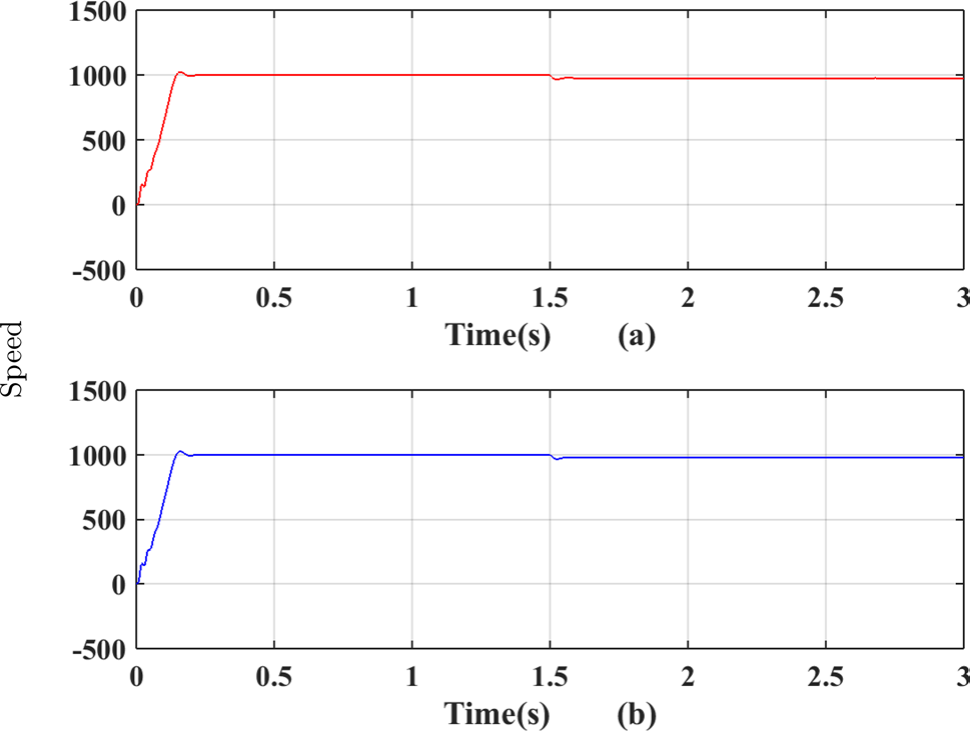
Speed (**a**) SVPWM, (**b**) RCMV-SVPWM.

The phase current waveforms for the conventional SVPWM and RCMV-SVPWM are shown in Fig. 12. As non-zero voltage vectors are used to eliminate common-mode voltage, the THD in current is increased from $2.3\%$ and $4.41\%$ in RCMV-SVPWM schemes. But it is still within acceptable limits. The developed toque is depicted in Fig. 13 and the motor speed in Fig. 14. As seen from the figures, they are identical in both modulation schemes.

### Experimental validations and discussion

**Figure 15:**
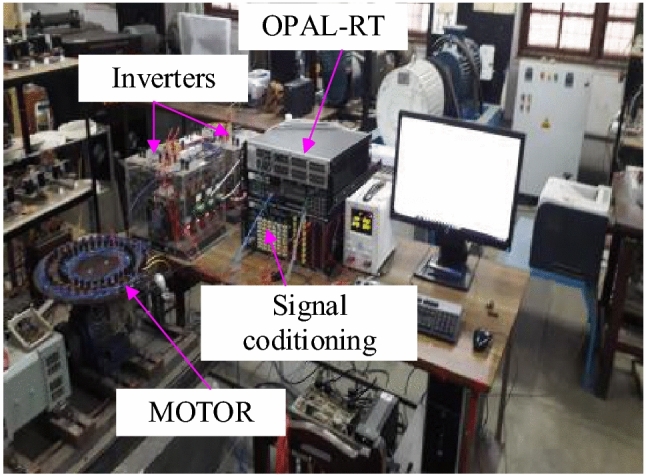
Exptsetup.pdf.

The proposed algorithm is verified using a test bench comprising of a dual three-phase inverter and dual three-phase induction machine, developed in the laboratory as shown in Fig. 15. The machine parameters are given in Appendix. Based on vector space decomposition theory, two PWM algorithms, namely the SVPWM and RCMV-SVPWM, are implemented on an FPGA-based OPAL-RT controller board (OP5660 along with OP8660). The low switching frequency of 2kHz is used for the dual three-phase machines, as they are used for high-power applications. The load is an asymmetrical six-phase squirrel cage motor having six poles made by rewinding the stator phases on the 36 stator slots of a 0.75-kW, 4-pole, three-phase motor. The performances of the proposed RCMV-SVPWM and SVPWM based on VSD techniques are compared. The phase current, line voltage, and CMV are measured without filtering by means of a TPS2014B Tektronix Oscilloscope. The common-mode voltage is measured between the -ve of the DC bus and the neutral of the machine.

**Figure 16:**
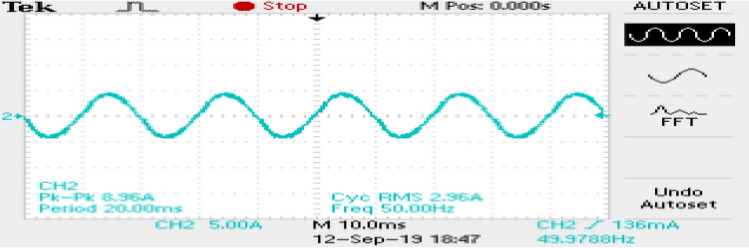
Line current (SVPWM).

**Figure 17:**
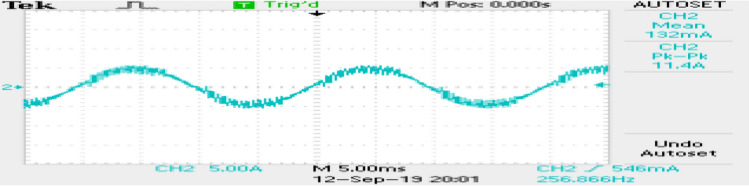
Line current (RCMV-SVPWM).

**Figure 18:**
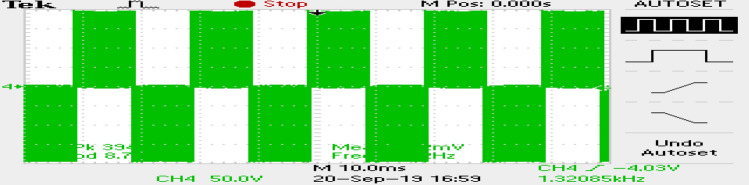
Line voltage (SVPWM).

**Figure 19:**
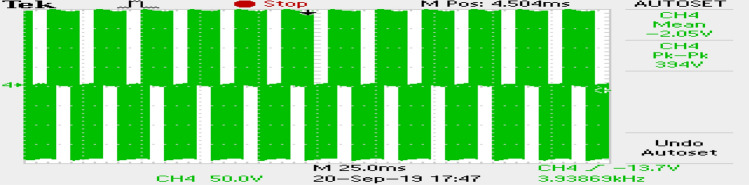
Line voltage (RCMV-SVPWM).

**Figure 20:**
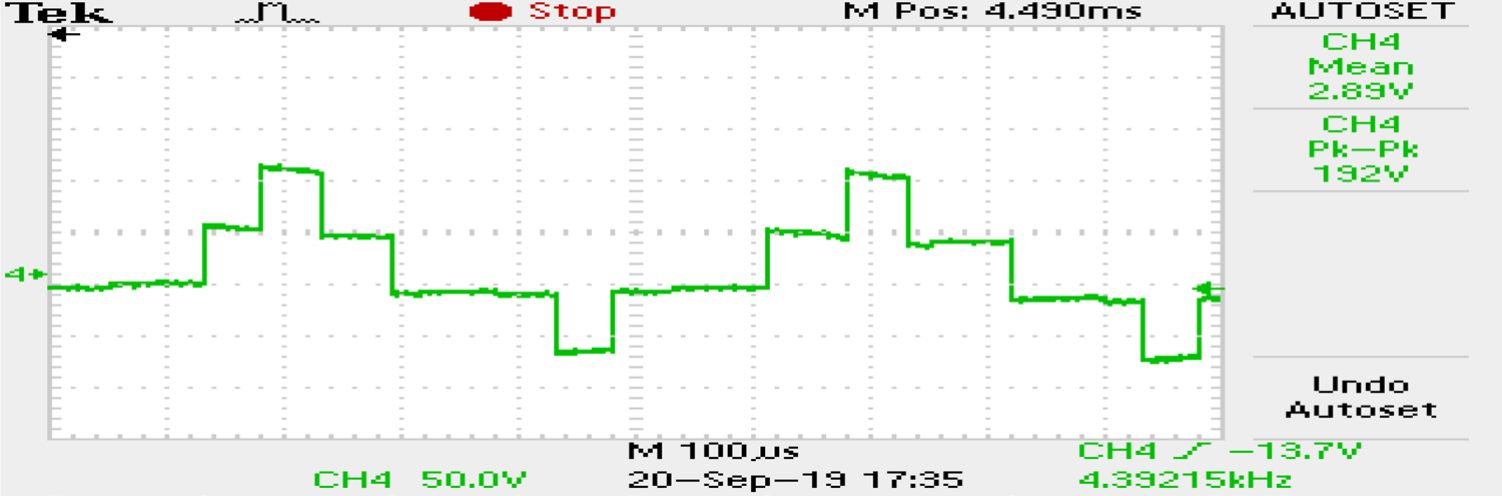
Common-mode voltage (SVPWM).

**Figure 21:**
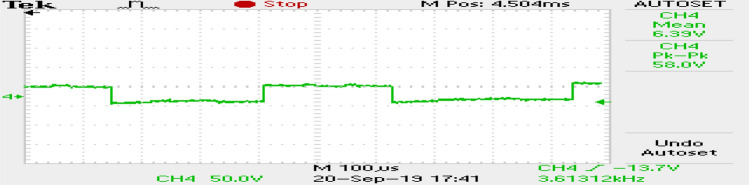
Common-mode voltage (RCMV-SVPWM).

The phase currents in the case of SVPWM and RCMV-SVPWM are depicted in Figs. 16 and 17. From the figure, it can be observed that the current waveforms are almost similar, measuring 2.3A and 2.35A, respectively. The line current THD is 2.1% and 3.8%. The line voltage waveforms are shown in Figs. 18 and 19 without much of a difference between them. With conventional SVPWM, the peak-to-peak CMV($V_n$) is 200V (Fig. 20) for a DC link voltage of 200V, much higher than the 66V (Fig. 21) for the same DC link voltage, obtained with the RCMV-SVPWM technique. Although there are spikes in CMV waveforms, they are largely due to the nonlinear behavior of the inverter and dead time. The reduction in common-mode voltage is the leading cause of the significant decrease in the high-frequency ripple content in the common-mode current. In summary, the proposed topology is able to reduce the common-mode voltage from half of the DC link voltage to one-sixth of the dc link voltage without hampering the other performance parameters, mainly current THD. A comparison between the two methods is mentioned in Table 4.

**Table 4:** Performance comparison.

Performance parameter	SVPWM	RCMV-SVPWM
CMV	Vdc/2	Vdc/6
No of switching in CMV	f	f/3
THD	2.1%	3.8%
RMS CMV	v	v/3

## Conclusion

In this paper, a space vector modulation technique based on vector space decomposition theory is presented for the reduction of common-mode voltage in dual three-phase machines supplied by a six-phase inverter. The performance of the dual three-phase induction motor drive is analyzed for the proposed RCMV-SVPWM and conventional SVPWM. The main contribution of the proposed work shows that the VSD theory can be used to reduce CMV to a great extent. From the results, it is seen that the proposed method not only reduces the amplitude of the common-mode voltage but also reduces the pulsations in the common-mode voltage. The current THD is also compared in both cases. Though the THD produced in the case of RCMV-SVPWM is slightly higher than that of SVPWM, it is still found that the THD produced in the case of RCMV-SVPWM is within the specified limits as per IEEE-519.

### Supplementary Information

Supplementary Information.

## Data Availability

The datasets used and/or analysed during the current study available from the corresponding author on reasonable request.

## List of symbols

$\lambda _s$: Stator flux linkage
$\lambda _{rr}$: Rotor self flux linkage
$\lambda _{rs}$: Rotor stator mutual flux linkage
$\lambda _{sr}$: Stator rotor mutual flux linkage
$\lambda _{ss}$: Stator self flux linkage
$CMV$: Common-mode voltage
$DTIM$: Dual three-phase induction machine
$$i_r$$: Rotor current
$$i_s$$: Stator current
$i_{\mu _1,\mu _2}$: Loss component in $\mu _1 -\mu _2$ plane
$i_{dr}$: d-axis rotor current
$i_{ds}$: d-axis stator current
$i_{qr}$: q-axis rotor current
$i_{qs}$: q-axis stator current
$i_{Z1,Z2}$: Zero sequence component in $Z_1-Z_2$ loss plane
$$L_{ms}$$: Magnetising inductance
$L_{sr}$: Stator rotor mutual inductance
$L_{ss}$: Stator self inductance
$RCMV$: Reduced common-mode voltage
$SVPWM$: Space vector pulse width modulation
$THD$: Total harmonic distortion
$$v_r$$: Rotor voltage
$$v_s$$: Stator voltage
$v_{ds}$: d-axis stator voltage
$v_{qs}$: q-axis stator voltage
$VSD$: Vector space decomposition
$VSI$: Voltage source inverter
$L_{lr}$: Rotor leakage inductance
$L_{ls}$: Stator leakage inductance
$L_{rr}$: Rotor self inductance
$L_{rs}$: Rotor stator mutual inductance
$R_{s1}, R_{s2}$: Stator resistances of the two three-phase winding sets
