# Supplementary material for: Modified SVPWM technique for CMV reduction in asymmetrical dual three phase induction machine drive
Source: Sci Rep. 2023 Dec 13;13:22108. doi: 10.1038/s41598-023-48339-3 (PMC10719323; doi:10.1038/s41598-023-48339-3)
Supplement: Supplementary file 1 — Supplementary Information. [file 41598_2023_48339_MOESM1_ESM.pdf]

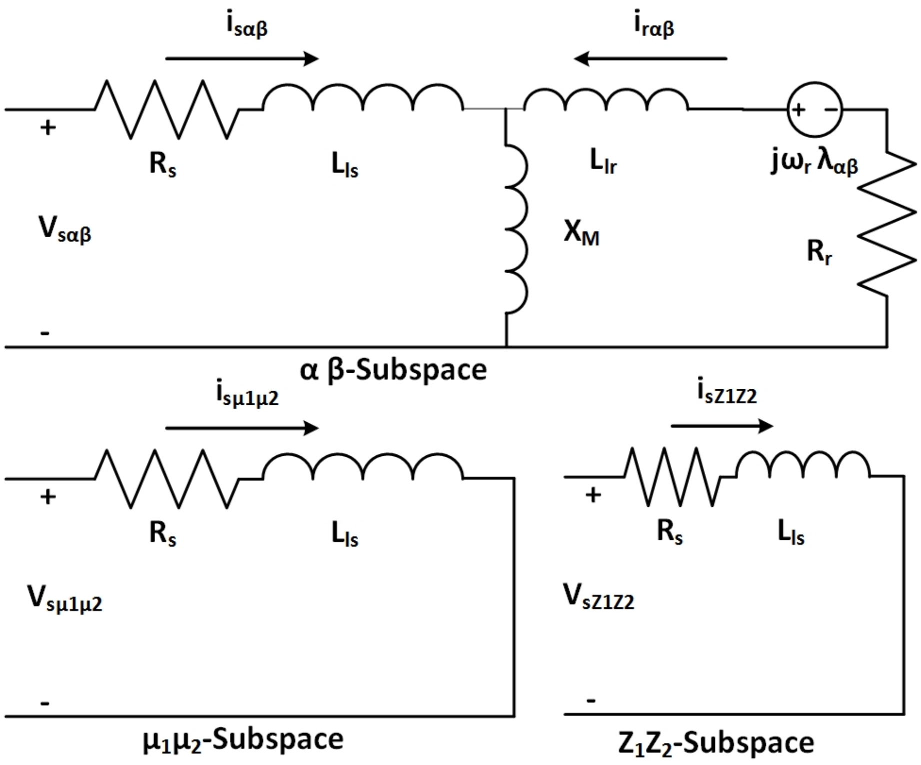

Figure 1: Equivalent circuit in $\alpha - \beta - \mu_1 - \mu_2 - z_1 - z_2$

1. Appendix :Modelling

Modeling of Dual Three Phase Induction Machine using VSD method The Equivalent circuit in $\alpha - \beta - \mu_1 - \mu_2 - z_1 - z_2$ is shown in Fig. 1

The stator voltage equation for a dual three-phase induction machine is,

$$\begin{aligned}
&[v_s] = [R_s][i_s] + p([\lambda_s]) \\
&= [R_s][i_s] + p([\lambda_{ss}] + [\lambda_{sr}]) \\
&= [R_s][i_s] + p([L_{ss}][i_s] + [L_{sr}][i_r])
\end{aligned} \tag{1}$$

The rotor voltage equation is,

$$\begin{aligned}
&[v_r] = [R_r][i_r] + p([\lambda_r]) \\
&= [R_r][i_r] + p([\lambda_{rr}] + [\lambda_{rs}]) \\
&= [R_r][i_r] + p([L_{rr}][i_r] + [L_{rs}][i_s])
\end{aligned} \tag{2}$$

The machine voltage equation in the $\alpha - \beta - \mu_1 - \mu_2 - z_1 - z_2$ subspace is presented below:

I) Machine Model in $\alpha - \beta$ subspace:
The stator voltage equation is

$$\begin{aligned} \begin{bmatrix} v_{ds} \\ v_{qs} \end{bmatrix} &= \begin{bmatrix} r_s & 0 \\ 0 & r_s \end{bmatrix} \begin{bmatrix} i_{ds} \\ i_{qs} \end{bmatrix} + \\ \frac{d}{dt} &\left\{ \begin{bmatrix} L_{ls} + 3L_{ms} & 0 \\ 0 & L_{ls} + 3L_{ms} \end{bmatrix} \begin{bmatrix} i_{ds} \\ i_{qs} \end{bmatrix} \right\} \\ + \frac{d}{dt} &\left\{ L_{ms} \begin{bmatrix} 3cos(\theta_r) & -3sin(\theta_r) \\ 3sin(\theta_r) & cos(\theta_r) \end{bmatrix} \begin{bmatrix} i_{dr} \\ i_{qr} \end{bmatrix} \right\} \end{aligned} \tag{3}$$

The rotor voltage equation is

$$\begin{aligned} \begin{bmatrix} 0 \\ 0 \end{bmatrix} &= \begin{bmatrix} r_r & 0 \\ 0 & r_r \end{bmatrix} \begin{bmatrix} i_{dr} \\ i_{qr} \end{bmatrix} + \\ \frac{d}{dt} &\left\{ \begin{bmatrix} L_{lr} + 3L_{ms} & 0 \\ 0 & L_{lr} + 3L_{ms} \end{bmatrix} \begin{bmatrix} i_{dr} \\ i_{qr} \end{bmatrix} \right\} + \\ \frac{d}{dt} &\left\{ \frac{N_r}{N_s} L_{ms} \begin{bmatrix} 3cos(\theta_r) & 3sin(\theta_r) \\ -3sin(\theta_r) & cos(\theta_r) \end{bmatrix} \begin{bmatrix} i_{ds} \\ i_{qs} \end{bmatrix} \right\} \end{aligned} \tag{4}$$

II) Motor model in $\mu_1 - \mu_2$ subspace:
The stator equation is

$$\frac{d}{dt} \begin{bmatrix} i_{z1s} \\ i_{z2s} \end{bmatrix} = \begin{bmatrix} -\frac{r_s}{L_{ls}} & 0 \\ 0 & -\frac{r_s}{L_{ls}} \end{bmatrix} \begin{bmatrix} i_{z1s} \\ i_{z2s} \end{bmatrix} + \begin{bmatrix} -\frac{1}{L_{ls}} & 0 \\ 0 & -\frac{1}{L_{ls}} \end{bmatrix} \begin{bmatrix} v_{z1s} \\ v_{z2s} \end{bmatrix} \tag{5}$$

The rotor equation is

$$\frac{d}{dt} \begin{bmatrix} i_{z1r} \\ i_{z2r} \end{bmatrix} = \begin{bmatrix} -\frac{r_r}{L_{lr}} & 0 \\ 0 & -\frac{r_r}{L_{lr}} \end{bmatrix} \begin{bmatrix} i_{z1r} \\ i_{z2r} \end{bmatrix} \tag{6}$$

III) Machine model in $z_1 - z_2$ subspace:
The stator equation is

$$\frac{d}{dt} \begin{bmatrix} i_{01s} \\ i_{02s} \end{bmatrix} = \begin{bmatrix} -\frac{r_s}{L_{ls}} & 0 \\ 0 & -\frac{r_s}{L_{ls}} \end{bmatrix} \begin{bmatrix} i_{01s} \\ i_{02s} \end{bmatrix} + \begin{bmatrix} -\frac{1}{L_{ls}} & 0 \\ 0 & -\frac{1}{L_{ls}} \end{bmatrix} \begin{bmatrix} v_{01s} \\ v_{02s} \end{bmatrix} \tag{7}$$

The rotor equation is

$$\frac{d}{dt} \begin{bmatrix} i_{01r} \\ i_{02r} \end{bmatrix} = \begin{bmatrix} -\frac{r_r}{L_{lr}} & 0 \\ 0 & -\frac{r_r}{L_{lr}} \end{bmatrix} \begin{bmatrix} i_{01r} \\ i_{02r} \end{bmatrix} \tag{8}$$

In the above formulation, the stator mutual leakage inductance is neglected, and therefore equations (5),(6),(7), and (8) have the same parameters. If mutual leakage inductance is considered, it will lead to a different formulation, but it does not contribute to the machine excitation. Hence, it will not affect the machine performance [**?**]. All the components responsible for electro-mechanical energy conversion lie on the d-q subspace, and non-electro-mechanical components belong to $z_1 - z_2$ and $0_1 - 0_2$ subspace. Thus, the dynamic equations of

the machine are completely decoupled. So it is easy for complex control strategy implementation, which is not in the case of conventional $d-q$ modeling. Further, rotor variables are transformed into stationary reference frames by applying the rotational transformation. The transformation matrix used is given as follows:

$$[T_r^s] = \begin{bmatrix} cos(\theta_r) & -sin(\theta_r) \\ sin(\theta_r) & cos(\theta_r) \end{bmatrix} \tag{9}$$

The combined stator and rotor equations in stationary reference frame obtained after applying the above transformation to equations (3) and (4) are:

$$\begin{bmatrix} v_{ds}^s \\ v_{qs}^s \\ 0 \\ 0 \end{bmatrix} = \begin{bmatrix} r_s + L_s p & 0 & M_p & 0 \\ 0 & r_s + L_s p & 0 & M_p \\ M_p & \omega_r M & r_r + L_r p & \omega_r L_r \\ -\omega_r M & M_p & -\omega_r L_r & r_r + L_r p \end{bmatrix} \begin{bmatrix} i_{ds}^s \\ i_{qs}^s \\ i_{dr}^s \\ i_{qr}^s \end{bmatrix} \tag{10}$$

where, $p = \frac{d}{dt}$
$L_s = L_{ls} + 3L_{ms}$
$L_r = L_{lr} + 3L_{ms}$
$M = 3L_{ms}$

The torque developed in the machine is given by

$$T_e = \frac{1}{2}[i]^T(\frac{\partial}{\partial \theta_r}[L])[i] \tag{11}$$

which is simplified as

$$\begin{aligned} T_e &= 3L_{ms}[i_{qs}^s(r_{dr}^r cos(\theta_r) - r_{qr}^r sin(\theta_r)) \\ &-i_{ds}^s(r_{dr}^r sin(\theta_r) + r_{qr}^r cos(\theta_r))] \\ &= 3L_{ms}\frac{P}{2}(i_{qs}^s.i_{dr}^s - i_{ds}^s.i_{qr}^s) \end{aligned} \tag{12}$$

where P, is the number poles in the machine.
